# Interventions involving young people for health equity targeting the fossil fuel industry’s practices and products: A realist review protocol

**DOI:** 10.1371/journal.pone.0356102

**Published:** 2026-08-13

**Authors:** Thilagawathi Abi Deivanayagam, Sandie-Marie Szawlowski, Sufiyan Abdul-Qayum, Araceli Camargo, Angela Fonso, Rachel Isba, Shweta Narayan, Audrey Prost, May van Schalkwyk, Delan Devakumar

**Affiliations:** 1 Institute for Global Health, University College London, London, United Kingdom; 2 Clean Air Southall and Hayes, London, United Kingdom; 3 The Centric Lab, United Kingdom; 4 Lancaster Medical School, Lancaster University, United Kingdom; 5 Alder Hey Children’s NHS Foundation Trust, Liverpool, Merseyside, United Kingdom; 6 Global Climate and Health Alliance, Richmond, California, United States of America; 7 Centre for Pesticide Suicide Prevention, University of Edinburgh, United Kingdom; 8 School of Social and Political Science, University of Edinburgh, United Kingdom; Wuhan University of Technology, CHINA

## Abstract

**Background:**

The fossil fuel industry’s practices and products create wide‑ranging harms to human health, with disproportionate impacts on communities already affected by structural disadvantage. Young people, particularly adolescents, are among those most affected by these harms, yet they are often excluded from health and climate decision-making. At the same time, youth-led initiatives to counter the fossil fuel industry’s practices and products are increasing globally. Despite this momentum, little is known about what interventions involving adolescents exist, how they work, and under what circumstances they may reduce health inequities driven by the fossil fuel industry. This study protocol aims to address this gap by conducting a realist review to explore how interventions involving adolescents can counter industry practices and products. It takes a structural racism lens to examine how inequities shape both exposure to harm and opportunities for participation. The review seeks to identify relevant interventions, understand the mechanisms through which they produce change, and examine the contexts that enable or hinder their effectiveness.

**Methods:**

The study follows established realist review standards and draws on diverse sources of evidence, including published research and grey literature. Adolescents and a multidisciplinary expert steering group have contributed to shaping the review questions and will continue to be involved throughout the process. The review will generate explanations about how interventions work by developing context–mechanism–outcome configurations. These explanations will be refined through iterative synthesis and engagement with advisory groups.

**Discussion:**

The review is expected to produce a set of theories illustrating how adolescents’ participation can influence action against the fossil fuel industry to help reduce health inequities. The findings will inform public health practitioners, policymakers, organisations working with adolescents, and researchers seeking to design, adapt, or scale interventions that amplify adolescents’ role in addressing the fossil fuel industry’s health harms.

## Introduction

Fossil fuels like oil and gas, and the world’s continued reliance on them, are a major threat to human health – from extraction to processing, to combustion, and disposal. An estimated 2.5 million deaths annually are due to outdoor air pollution from burning fossil fuels [1]. Fossil fuels contribute to respiratory illnesses, cancers, cardiovascular diseases, and congenital diseases [2]. However, these harms are not distributed equally. Communities already facing structural disadvantage – particularly those on low incomes and racially minoritised people – experience disproportionate exposure to fossil fuel-related pollution and health risks [3]. From extraction sites often located near minoritised communities to the burden of air pollution, these inequities often fall on those least responsible for fossil fuel use, creating a cycle of harm that persists from cradle to grave [1]. The fossil fuel industry is also the driving force behind climate change, with 67% of greenhouse gases attributable to fossil fuel combustion. Climate change increases food insecurity, infectious diseases spread, heat-related illness, and deaths from climate-related disasters such as flooding, among other negative health consequences [1].

There is a growing body of literature examining how the practices and products of commercial actors impact health [4]. Despite strong scientific consensus on these harms and the availability of cleaner, more equitable energy alternatives, fossil fuel extraction projects continue to expand [5]. This is enabled by industry practices that block, delay and dilute policy similar to those adopted by the tobacco and other health-harming industries (HHIs) [6–9]. Practices include spreading doubt by undermining the independent evidence base and funding favourable research, as well as spreading misinformation and lobbying against regulations [10]. This results in national and international policies that align more with the business interests of the fossil fuel industry than with those of people and communities affected by its health impacts [11]. The health community is increasingly starting to recognise the fossil fuel industry as a commercial determinant of health (CDoH) [12]. These are defined as the strategies and approaches used by the private sector to promote products and choices that are detrimental to health [13]. Understanding the fossil fuel industry through a CDoH lens is critical because these industry practices are the primary drivers of continued fossil fuel dependence. Countering these practices is essential to achieving a global phase-out of the industry’s products (coal, oil, and gas).

The health community’s resistance to fossil fuels has never been stronger. In 2022, 192 health organisations worldwide, including the World Health Organization (WHO), have demanded a phase-out of fossil fuels by calling for an international fossil fuel non-proliferation treaty [14]. A 2023 open letter from organisations representing 46.3 million health workers worldwide also helped secure commitment to transition away from fossil fuels [15].

Children, defined as anyone under the age of 18 years, disproportionately suffer the health harms of fossil fuel use but are frequently excluded from climate decision-making [16,17]. The population we are interested in for this review are adolescents (aged 10–19 years old). The worsening threat of climate change affects all aspects of adolescents’ wellbeing [18]. Many feel disillusioned by inadequate action, [19] prompting resistance led by adolescents against fossil fuel projects, through tactics such as litigation, school strikes, and campaigns [20]. The UK Government [21] and United Nations (UN) policies [22] advocate for adolescents’ participation in both health and climate decision-making. The UK is also a signatory to the UN Convention on the Rights of the Child, a global treaty requiring children to participate in policymaking that affects them.

Health inequities persist, especially for racially minoritised children, who face greater harms from climate change [16] and more frequent exclusion from research and policy spaces [23,24]. Minoritised refers to individuals or populations (even numerical majorities) whose power is eroded by societal structures targeting their identity [25]. These inequities stem from structural racism – systems embedded in laws, institutions, and societal narratives that organise advantage and disadvantage [26]. Other intersecting factors such as class, gender, and disability can compound health inequities rising from climate change [3]. Structural racism also operates at the level of whose knowledge is recognised as legitimate; it influences the norms, resources, and cognitive habits that determine how knowledge is produced and valued [27]. Ensuring racialised adolescents’ meaningful involvement in policy and decision making is therefore essential not only because they are most affected, but also as an act of epistemic justice. Their lived experience offers insights necessary for equitable public health responses and for challenging the fossil fuel industry’s role in driving health inequities [18].

This protocol is for a realist review of interventions (programmes, campaigns or other initiatives) involving adolescents that counter the fossil fuel industry’s practices and products, with the outcome of addressing health inequity. Current health literature is skewed towards monitoring the health impacts of climate change, rather than evaluating the effectiveness and equity of interventions addressing them [28]. This review aims to identify existing interventions, explore their mechanisms of change, and inform public health responses that integrate young voices. A realist approach is well-suited for this task as it examines *how* interventions produce outcomes rather than simply asking “does it work”. By uncovering generative mechanisms and contextual factors, realist review can guide adaptation and scaling of interventions across settings [29]. Findings will inform a wider study to create an advocacy strategy addressing the fossil fuel industry’s role in driving climate-related health inequalities through a structural racism lens [30].

## Methods

### What is a realist review?

A realist review is a type of literature review that seeks to explain how, why, and for whom interventions work [31]. It aims to provide a causal understanding rather than simply assessing effectiveness by employing the Context–Mechanism–Outcome (CMO) framework (See Table 1) [33]. In other words, outcomes occur when mechanisms are triggered within particular contexts.

**Table 1:** Key definitions derived from Pawson et al.’s text on realist methodology [32].

Context	Context refers to the backdrop of the research, in this case, the conditions that enable or obstruct interventions. This might include the social, political, cultural, and economic factors that shape change. It can also include the geographic setting or funding sources.
Mechanism	The underpinning generative force that leads to intended or unintended outcomes, divided into two parts: • Resources offered by an intervention (including economical, material, emotional, social, or knowledge resources). • How people respond to those resources
Outcome	Intended or unintended impact resulting from the context-mechanism interaction.
Context-mechanism-outcome (CMO) configuration	CMO configuring is a heuristic used to generate causal explanations based on the evidence. It is a tool that helps articulate the context, mechanism and outcome, bringing us closer to developing and refining the theories that become the final product of the review.
Initial programme theory (IPT)	A hypothetical statement, often in the form of “If… then”, developed at the beginning of the review to explain how a programme or intervention is thought to work (or not) and for whom.

Grounded in the realist paradigm, this approach emphasises generative causation, i.e., understanding how programmes and policies produce outcomes through human decisions [34]. Analysis is theory-driven and iterative, aiming to develop and refine programme theories using evidence from diverse sources. These theories will articulate and critique the assumptions underlying intervention design and guide adaptation across settings [35].

Our review will follow Realist And Meta-narrative Evidence Syntheses: Evolving Standards (RAMESES) standards [36] and realist methodology, comprising five stages: (1) defining the review’s scope, (2) building initial programme theory, (3) systematic evidence search, (4) selection and appraisal and (5) extraction and synthesis [37].

### Who we are

The research team consists of a core group of two people (TAD and SS). TAD is the project lead and PhD student. SS is a PhD student and will be the second independent screener and data extractor. The project lead is supervised by DD, AP, and RI. A youth advisory board (YAB) made up of six adolescents who identify as racially minoritised aged 14–17 years from across England were invited to participate. A multidisciplinary expert steering group (ESG) has representation from parents, community-based organisations, experts by experience, researchers and public health professionals. These partners helped in shaping the initial review questions and reviewing the protocol. Moving forward, they will continue to help refine key criteria, interpret the findings, and distil the programme theories. Some of them join as authors on this protocol. Shared agreements on ways of working for the YAB and ESG were written at the start of the review and can be found in S1 File. The core group and supervisors will continue to meet monthly during the research process to review progress, develop procedures and troubleshoot. TAD will maintain correspondence with the YAB and ESG.

### Step 1. Define the review scope

According to RAMESES standards, the first step of a realist review is to clarify the scope and definitions of key concepts [32]. A preliminary search across PubMed, GeoBase and Scopus helped develop a sense of the size of the evidence base and inform the scope and definitions. The root terms were ‘climate change’, ‘young people’, ‘fossil fuel’, and ‘advocacy/intervention’, with no restrictions on date or methods. From this, we refined definitions and identified gaps in interventions involving minoritised adolescents addressing fossil fuel-related health inequities.

We define adolescents as 10–19-year-olds, according to the UN definition [38]. The fossil fuel industry in this review is defined as the corporate entities engaged in the extraction, transport, refinement, and sale of oil, coal, and gas, and their derivative products. This includes their owners (private or state), trade bodies, and those entities acting on their behalf while in receipt of their funding, such as think tanks, public relations, and legal firms [39]. Interventions of interest include advocacy initiatives, litigation, activism, campaigns, policymaking, or strategies that involve adolescents. We acknowledge that levels of involvement or participation will vary across interventions. Studies will not be excluded based on low levels of participation alone. Instead, we will include interventions where adolescents’ involvement corresponds to Rung five and above (consulted and informed) on Hart’s ladder of children’s participation [40]. The ladder then will be used analytically to classify and interpret degrees of involvement, and ultimately power-sharing during data extraction and synthesis, rather than a strict exclusion criterion.

Governments can introduce policies to reduce harm, but the fossil fuel industry repeatedly blocks, weakens, and delays such policies through what is known as *corporate political activity* (CPA). These are “practices to secure preferential treatment and/or prevent, shape, circumvent or undermine public policies in ways that further corporate interests” [41]. In this review, we will include interventions that counter industry CPA both directly and indirectly. This means including, for example, campaigns advocating for clean air, renewable energy initiatives, or education programmes that raise awareness of the industry’s harms and build collective power. While these do not directly target fossil fuel companies or their products, they are helping to counter the narratives and sociopolitical norms “framed” by the industry that serve to normalise their products and play down the harms. Therefore, any interventions that have an element that counters the fossil fuel industry’s CPA will be included.

Addressing health inequities – which stem from wider political and social inequities – requires building social and policy movements capable of challenging the structural drivers of harm. Interventions that counter industry CPA form part of a wider *movement ecosystem*, in which community actors, youth groups, civil society organisations, and social movements collectively work to resist the industry’s practices and influence. Movement ecosystems provide the infrastructure, relationships, and strategic capacities needed for policy and systems change, as highlighted in the social movement literature [42,43]. Recognising these interventions as elements of a broader movement landscape reinforces the importance of applying a CPA lens in this review, as it allows us to capture how collective action, organising, and movement-driven pressure contribute to challenging the health inequities produced by the fossil fuel industry.

This review uses a structural racism lens, [44] acknowledging racial discrimination in policymaking, that racially minoritised people experience higher exposure to health harms, [45] and their exclusion from research, decision-making and environmental movements [3]. This lens necessitates most affected people’s participation in research questions, methods, and outputs (see ‘Who we are’) [46]. Interventions that are participatory, anti-racist, or rooted in social movements will be prioritised in the search. Studies referencing health inequities linked to racism will rank higher in relevance during appraisal. However, we acknowledge that many primary studies measure disparities without explicitly naming racism [47]. Data extraction and synthesis will note these limitations. Devakumar and colleagues’ conceptual model on racism and health will be referred to during data synthesis [48]. This model is well-suited to the review because it attributes health inequities to macro-level power hierarchies embedded across society. Applying this model allows us to frame the health harms of fossil fuels as an intergenerational health crisis, driven by commercial extractivism and structural racism [3].

The realist review questions emerging from the preliminary search and discussion between the researchers, ESG, YAB, and supervisory team were:

1. **What interventions (programmes, campaigns, or other initiatives) exist involving adolescents to address health inequities driven by the fossil fuel industry’s practices and products?** (mechanism and outcomes)
2. **What are the mechanisms of change of these interventions?** (mechanism)
3. **What are the circumstances that influence (e.g., enable or impede) these interventions’ functioning?** (context)

### Step 2. Build initial programme theories

Draft initial programme theories (IPTs) were generated from early searches and can be found in S2 File. Draft theories were refined through consensus building workshops with the YAB and ESG. Participants discussed intervention examples, and critiqued and ranked IPTs based on lived experience and prior work. The IPTs will guide evidence searching and synthesis.

To structure theory development, alongside Devakumar’s model [48], we will also refer to the MPOWER+ framework, adapted from tobacco control policy [49]. MPOWER+ has seven domains: monitor, protect, offer support, warn, enforce bans, raise taxes, and restrict undue commercial influence. The framework offers a systematic approach for public health action against fossil fuel-related harms, explicitly taking a CDoH lens. The original MPOWER included coordinated, evidence-based policies that significantly reduced smoking rates globally [50]. A key lesson from tobacco control was the adoption of Article 5.3 of the WHO Framework Convention on Tobacco Control (FCTC) [51]. This called for the protection of public health policies from the vested interests of the tobacco industry, supported by regulations that restrict industry influence. Where implemented, it helped to reduce the tobacco industry’s access to policymaking spaces and increase transparency. This Article is widely recognised as essential to making progress in addressing the health harms from tobacco. Drawing on lessons from tobacco control, where understanding industry practices was central to effective action, MPOWER+ may provide a benchmark for examining interventions seeking to counter these influences [52].

### Step 3. Evidence searches – identification of data

The search strategy was developed by TAD and reviewed by the ESG and supervisory team (See S3 File). Review papers, qualitative and quantitative studies, evaluations, reports, and strategy documents will be identified. No date, geographic, or language restrictions will apply. Non-English documents will be translated using Google Translate.

Data sources will include:

1. Databases: PubMed, Global Health, Scopus, Web of Science, and GEOBASE.
2. Citation searching from primary studies and reviews.
3. Purposive hand searching of websites and organisational reports such as UNICEF, WHO, and Fridays for Future. Only reports that provide evidence to inform the development of CMOs will be included.
4. Input will be sought from the supervisory team and ESG to identify additional relevant publications, guidelines, and policies.

Further searches may be needed as the review progresses to support or refute the initial or emerging programme theories. All retrieved records will be imported into EndNote [53] for organisation and de-duplication, before transferring to Rayyan [54] to facilitate title and abstract screening.

### Step 4. Selection and appraisal

Screening will follow Preferred Reporting Items for Systematic Reviews and Meta-Analyses (PRISMA) guidance [55]. Inclusion and exclusion criteria are summarised in Table 2 and may need refinement as the review progresses [35]. Given the emerging nature of the literature, we will apply these criteria broadly and err on the side of inclusion rather than exclusion at this stage if the reviewers feel the document will aide theory-building. Two reviewers (TAD and SS) will independently screen papers first by title and abstract. For grey literature, the executive summary will be read in full.

**Table 2:** Review inclusion and exclusion criteria.

Inclusion criteria	
Population	• Adolescents. • Adolescents who are racially minoritised or another similar descriptor. • If age is not stated, but there are references to adolescents, youth or young people, include at the first stage.
Intervention	• Any intervention (programme, campaign, or initiative) that involves one of the above groups at any stage of development, implementation, or evaluation to counter the fossil fuel industry’s practices and products. • Adolescents are consulted and informed (Rung five in Hart’s ladder of child participation).
Comparator	None.
Outcome	• Outcomes of interest will depend on the intervention but could include any measurable impact (intended or unintended) on health or health (in)equity, e.g., by racism, classism, disability or gender injustice. • Studies are not required to report comparisons of health outcomes between population groups. • Interim outcomes on the pathway to health such as wellbeing, self-esteem, skills, critical science literacy, and health literacy will be documented.
Design	No restriction.
Location	Worldwide.
**Exclusion criteria**	
	Interventions that are broadly related to climate change, and not specific to countering the fossil fuel industry’s products and practices directly or indirectly.
	Interventions that do not involve adolescents.

Two reviewers will undertake quality appraisal. In line with the realist approach, quality appraisal will be completed according to three criteria: relevance, richness, and rigour [56]. Criteria for ranking relevance developed with the ESG and supervisory team are shown in Table 3. If a study has high or moderate relevance, the full text will be appraised for richness and rigour.

**Table 3:** Criteria to rank likely relevance of study to guide theory development.

High relevance	• Relates to action taken by an individual or group of people challenging the fossil fuel industry’s practices or products. • Refers to health inequity, specifically due to gender, racial, disability or class injustice. • Describes how adolescents are involved in design, implementation and/or evaluation. • Describes the resources available or provided to practitioners to involve adolescents or for adolescents to lead. • Describes the perspectives of adolescents and the factors affecting their involvement. • Relates to structural racism in policy and decision making.
Moderate relevance	• Relates to adolescents and their involvement in climate justice-related activities. • Describes the health harms of the fossil fuel industry or the health benefits for acting. • Relates to addressing the health impacts of climate change in adolescents. • Refers to health inequity at the spatial level or other.
Low relevance	• Relates to adolescents’ involvement in climate change-related actions more broadly. • Describes the health impacts of climate change in general
No relevance	Does not meet any of the above criteria.

Richness is whether the studies describe theories or concepts or describe the set of efforts needed to enact change [36]. Criteria from the existing literature [57] will be adapted to assess richness. Rigour refers to trustworthiness and credibility of the document [36], i.e., does the evidence support the conclusions made from it? The realist approach rejects the traditional ‘hierarchy of evidence’ whereby randomised controlled trials (RCTs) sit atop and opinion pieces underneath: valuable causal insights could be missed if seemingly poor-quality studies are ignored. We will therefore consider evidence of ‘lesser quality’ if they are relevant for identifying and developing programme theories for our study. Rigour will be assessed using RAMESES standards [36].

In this review, any studies and grey literature that describe the context of adolescents being involved in implementation plans and strategies challenging the fossil fuel industry, explore mechanisms and outcomes, and are in alignment with the inclusion criteria and search terms will be appraised. We will use a flow diagram for realist reviews [58] to show the process of selecting studies (S4 File). Any design limitations will be documented in an appraisal form adapting an existing template (S5 File) [59].

### Step 5. Extraction and synthesis

Two reviewers (TAD and SS) will re-read the full texts and complete the data extraction, which will be discussed with the rest of the team in our regular meetings. Data extraction and synthesis for realist review is guided and RAMESES standards [36] Information will be extracted into a bespoke data extraction form (S6 File) capturing:

- Context (geographic setting, study type, target population, sampling)
- Intervention (activities, content, delivery mode, duration, staff)
- Outcomes (definitions, measures, time frame, findings)
- Notes on structural racism and equity considerations

Synthesis refers to the explanatory pursuit that will lead to an understanding of how, for whom and under what circumstances interventions involving adolescents in challenging the health inequalities generated by the fossil fuel industry work. Since realist reviews begin with theories and end with more refined theories, this step intends to translate data analysis into a refined ‘realist theory’. Methodological recommendations from the literature will be used and qualitative evidence will be synthesised by applying a realist approach to thematic analysis [60]. We will use retroductive reasoning [61] to refine CMO configurations and develop the final ‘realist theory’. Visual summaries of CMO configurations will be produced and validated through workshops with the YAB and ESG. These insights will inform future research related to interventions involving adolescents – a population disproportionately impacted by the harms of the fossil fuel industry. The outputs will be of value to funders, practitioners, policymakers, and researchers interested in creating evidence-informed solutions to counter the health harms driven by the industry [62].

The protocol is registered with PROSPERO: ID number 1267252. The completed PRISMA protocol checklist can be found in S7 File) [55]. No ethical approval is required as no primary data will be collected or analysed for the realist review. However, ethical approval has been obtained from the University College London Research Ethics Committee for the wider project that this review is part of (ID number: 1253).

### Study status

Study start date: October 2024

Expected end date: April 2027

At the time of writing, the study status is as follows:

1. Define the review scope: started.
2. Build initial programme theories: started.
3. Evidence searches: started.
4. Selection and appraisal: not started.
5. Data extraction and synthesis: not started.

Data collection (evidence searches, selection and appraisal) is aimed to be completed by April 2026. Results for the realist review are expected in July 2026. Dissemination will be completed by April 2027.

## Discussion

A key strength of the study is that a comprehensive search strategy has been created to identify the most relevant literature. This includes systematic searches of academic databases, grey literature, and complementary techniques such as citation searching and snowballing. Youth advisors and an interdisciplinary panel of experts – spanning policy, advocacy, and research – have contributed to the development of this study, ensuring diverse perspectives are embedded throughout. However, engaging multiple stakeholder groups may present challenges in achieving consensus, particularly when shaping, refining, and prioritising programme theories that reflect complex social and political determinants.

Findings from the review will be published in a peer-review journal and shared via a report to the funder (NIHR), blogs, conference presentations, and social media (TikTok, Instagram, and LinkedIn posts). Existing contacts via the UK Faculty of Public Health and NHS are further avenues for dissemination. We will work with the UCL Communications team to produce a press release. Outputs aim to inform funding, policy, research, and collaboration with adolescents to address the health harms of fossil fuels. We intend for the review to provide an evidence base for shaping an inclusive response against the main driver of climate change.

## Supporting information

S1 File Shared assumptions and agreements. (DOCX)

S2 File Draft initial programme theories. (DOCX)

S3 File Search strategy. (DOCX)

S4 File Flowchart. (DOCX)

S5 File Quality appraisal form. (DOCX)

S6 File Data extraction form. (DOCX)

S7 File PRISMA-P Checklist. (DOCX)
