# Supplementary material for: Interventions involving young people for health equity targeting the fossil fuel industry’s practices and products: A realist review protocol
Source: PLoS One. 2026 Aug 13;21(8):e0356102. doi: 10.1371/journal.pone.0356102 (PMC13472360; doi:10.1371/journal.pone.0356102)
Supplement: Supplementary file 1 [file pone.0356102.s001.docx]

**Shared agreements for Youth Advisory Board and Expert Steering Group**

1. **Come as you are**. We are not looking for perfection.
2. **Power and privilege**. We encourage awareness of personal privileges, and to be aware of how much space you take up accordingly.
3. "**Make Space" or "Take Space**". Demonstrate respect for others by recognising how often, much, and loud you’re speaking and whether or not you’re dominating conversation.
4. **“One mic”.** One person speaks at a time.
5. **Care**. Prioritise care for each other.
6. **Curiosity**. Ask questions. Curiosity to be met without judgement.
7. **Lived experience and trauma**. These topics are sensitive and real stories. Be respectful.
8. **Confidentiality.** We operate a safe space, and a closed space.
9. **Consent**. If you wish to discuss anything shared in this space outside of it, please ask first or speak with the project lead.
10. **Share** **trigger or content warnings** before sharing sensitive information.
11. **Learn from mistakes**. Prioritise progress.
12. **Declare conflict of interest.** Complete the form and declare any changes.
