# Supplementary material for: Interventions involving young people for health equity targeting the fossil fuel industry’s practices and products: A realist review protocol
Source: PLoS One. 2026 Aug 13;21(8):e0356102. doi: 10.1371/journal.pone.0356102 (PMC13472360; doi:10.1371/journal.pone.0356102)
Supplement: Supplementary file 2 [file pone.0356102.s002.docx]

**Draft PubMed search strategy**

| Search number | Query | Search details | Results |
| --- | --- | --- | --- |
| #1 | **"fossil fuel industry" or "fossil fuel company" or "coal industry" or "fossil fuel" or "oil industry" or "gas industry" or " coal mine" or "coalmine" or "fracking" or "gas flaring" or "oil field" or "gasworks" or "coal mining" or "extractive industry" or "polluting industry" or "petrochemical" or "oil and gas"** | "fossil fuel industry"[All Fields] OR (("fossil fuels"[Supplementary Concept] OR "fossil fuels"[All Fields] OR "fossil fuel"[All Fields] OR "fossil fuels"[MeSH Terms] OR ("fossil"[All Fields] AND "fuels"[All Fields]) OR ("fossil"[All Fields] AND "fuel"[All Fields])) AND ("companies"[All Fields] OR "company"[All Fields] OR "company s"[All Fields] OR "companys"[All Fields])) OR "coal industry"[All Fields] OR "fossil fuel"[All Fields] OR "oil industry"[All Fields] OR "gas industry"[All Fields] OR "coal mine"[All Fields] OR "coalmine"[All Fields] OR "fracking"[All Fields] OR "gas flaring"[All Fields] OR "oil field"[All Fields] OR "gasworks"[All Fields] OR "coal mining"[All Fields] OR "extractive industry"[All Fields] OR "polluting industry"[All Fields] OR "petrochemical"[All Fields] OR "oil and gas"[All Fields] | 38,628 |
| #2 | **(advocacy or policy or strategy or intervention or campaign or litigat* or "legal action" or activism or action)** | ("advocacies"[All Fields] OR "advocacy"[All Fields] OR "advocacy s"[All Fields] OR ("policy"[MeSH Terms] OR "policy"[All Fields] OR "policies"[All Fields] OR "policy s"[All Fields]) OR ("strategie"[All Fields] OR "strategies"[All Fields] OR "strategy"[All Fields] OR "strategy s"[All Fields]) OR ("intervention s"[All Fields] OR "interventions"[All Fields] OR "interventive"[All Fields] OR "methods"[MeSH Terms] OR "methods"[All Fields] OR "intervention"[All Fields] OR "interventional"[All Fields]) OR ("campaign"[All Fields] OR "campaign s"[All Fields] OR "campaigned"[All Fields] OR "campaigner"[All Fields] OR "campaigners"[All Fields] OR "campaigning"[All Fields] OR "campaigns"[All Fields]) OR "litigat*"[All Fields] OR "legal action"[All Fields] OR "activism"[All Fields] OR ("action"[All Fields] OR "action s"[All Fields] OR "actions"[All Fields])) | 14,452,115 |
| 3 | **(structural or political or "cultural safety" or anti-racis* or decolonial* or equit* or justice or "rights-based" or coproduc* or co-produc* or collective or "action research" or "participatory research" or "participatory action research" or "youth participatory action research")** | ("structural"[All Fields] OR "structurally"[All Fields] OR "structurals"[All Fields] OR "structuration"[All Fields] OR "structurations"[All Fields] OR "structure"[All Fields] OR "structure s"[All Fields] OR "structured"[All Fields] OR "structures"[All Fields] OR "structuring"[All Fields] OR ("political"[All Fields] OR "politically"[All Fields] OR "politics"[MeSH Terms] OR "politics"[All Fields] OR "politic"[All Fields]) OR "cultural safety"[All Fields] OR "anti racis*"[All Fields] OR "decolonial*"[All Fields] OR "equit*"[All Fields] OR ("justice s"[All Fields] OR "social justice"[MeSH Terms] OR ("social"[All Fields] AND "justice"[All Fields]) OR "social justice"[All Fields] OR "justice"[All Fields] OR "justices"[All Fields]) OR "rights-based"[All Fields] OR "coproduc*"[All Fields] OR "co produc*"[All Fields] OR ("collective"[All Fields] OR "collectiveness"[All Fields] OR "collectives"[All Fields] OR "collectivities"[All Fields] OR "collectivity"[All Fields]) OR "action research"[All Fields] OR "participatory research"[All Fields] OR "participatory action research"[All Fields] OR "youth participatory action research"[All Fields]) | 4,026,935 |
| 4 | **#2 AND #3** | ("advocacies"[All Fields] OR "advocacy"[All Fields] OR "advocacy s"[All Fields] OR ("policy"[MeSH Terms] OR "policy"[All Fields] OR "policies"[All Fields] OR "policy s"[All Fields]) OR ("strategie"[All Fields] OR "strategies"[All Fields] OR "strategy"[All Fields] OR "strategy s"[All Fields]) OR ("intervention s"[All Fields] OR "interventions"[All Fields] OR "interventive"[All Fields] OR "methods"[MeSH Terms] OR "methods"[All Fields] OR "intervention"[All Fields] OR "interventional"[All Fields]) OR ("campaign"[All Fields] OR "campaign s"[All Fields] OR "campaigned"[All Fields] OR "campaigner"[All Fields] OR "campaigners"[All Fields] OR "campaigning"[All Fields] OR "campaigns"[All Fields]) OR "litigat*"[All Fields] OR "legal action"[All Fields] OR "activism"[All Fields] OR ("action"[All Fields] OR "action s"[All Fields] OR "actions"[All Fields])) AND ("structural"[All Fields] OR "structurally"[All Fields] OR "structurals"[All Fields] OR "structuration"[All Fields] OR "structurations"[All Fields] OR "structure"[All Fields] OR "structure s"[All Fields] OR "structured"[All Fields] OR "structures"[All Fields] OR "structuring"[All Fields] OR ("political"[All Fields] OR "politically"[All Fields] OR "politics"[MeSH Terms] OR "politics"[All Fields] OR "politic"[All Fields]) OR "cultural safety"[All Fields] OR "anti racis*"[All Fields] OR "decolonial*"[All Fields] OR "equit*"[All Fields] OR ("justice s"[All Fields] OR "social justice"[MeSH Terms] OR ("social"[All Fields] AND "justice"[All Fields]) OR "social justice"[All Fields] OR "justice"[All Fields] OR "justices"[All Fields]) OR "rights-based"[All Fields] OR "coproduc*"[All Fields] OR "co produc*"[All Fields] OR ("collective"[All Fields] OR "collectiveness"[All Fields] OR "collectives"[All Fields] OR "collectivities"[All Fields] OR "collectivity"[All Fields]) OR "action research"[All Fields] OR "participatory research"[All Fields] OR "participatory action research"[All Fields] OR "youth participatory action research"[All Fields]) | 1,587,167 |
| #5 | **"Youth-led" or "youth led" or "Young people" or "young person" or Youth or adolescen* or children "Youth involvement" or "Youth engage*" or "youth participat*" or "youth empowerment" or "youth mobilization" or "youth mobilisation" or "youth experience" or "children and young people" or "child-led" or "child led" or "child involvement" or "child-centred" or "child centred" or "youth-informed" or "child informed" or "primary school*" or "secondary school*" or "high school*”** | (("Youth-led"[All Fields] OR "Youth-led"[All Fields] OR "Young people"[All Fields] OR "young person"[All Fields] OR ("adolescent"[MeSH Terms] OR "adolescent"[All Fields] OR "youth"[All Fields] OR "youths"[All Fields] OR "youth s"[All Fields]) OR "adolescen*"[All Fields] OR ("child"[MeSH Terms] OR "child"[All Fields] OR "children"[All Fields] OR "child s"[All Fields] OR "children s"[All Fields] OR "childrens"[All Fields] OR "childs"[All Fields])) AND "Youth involvement"[All Fields]) OR "youth engage*"[All Fields] OR "youth participat*"[All Fields] OR "youth empowerment"[All Fields] OR "youth mobilization"[All Fields] OR (("adolescent"[MeSH Terms] OR "adolescent"[All Fields] OR "youth"[All Fields] OR "youths"[All Fields] OR "youth s"[All Fields]) AND ("mobilisation"[All Fields] OR "mobilisations"[All Fields] OR "mobilise"[All Fields] OR "mobilised"[All Fields] OR "mobiliser"[All Fields] OR "mobilisers"[All Fields] OR "mobilises"[All Fields] OR "mobilising"[All Fields] OR "mobilization"[All Fields] OR "mobilizations"[All Fields] OR "mobilize"[All Fields] OR "mobilized"[All Fields] OR "mobilizer"[All Fields] OR "mobilizers"[All Fields] OR "mobilizes"[All Fields] OR "mobilizing"[All Fields])) OR "youth experience"[All Fields] OR "children and young people"[All Fields] OR "child-led"[All Fields] OR "child-led"[All Fields] OR "child involvement"[All Fields] OR "child-centred"[All Fields] OR "child-centred"[All Fields] OR "youth-informed"[All Fields] OR "child informed"[All Fields] OR "primary school*"[All Fields] OR "secondary school*"[All Fields] OR ("high"[All Fields] AND "school*"[All Fields]) | 1,596,461 |
| #6 | **health or "health outcomes" or "child health" or "public health" or "health inequalit*" or "mental health" or "health inequit*" or "health disparit*" or disease or illness or wellbeing or well-being** | "health"[MeSH Terms] OR "health"[All Fields] OR "health s"[All Fields] OR "healthful"[All Fields] OR "healthfulness"[All Fields] OR "healths"[All Fields] OR "health outcomes"[All Fields] OR "child health"[All Fields] OR "public health"[All Fields] OR "health inequalit*"[All Fields] OR "mental health"[All Fields] OR "health inequit*"[All Fields] OR "health disparit*"[All Fields] OR "disease"[MeSH Terms] OR "disease"[All Fields] OR "diseases"[All Fields] OR "disease s"[All Fields] OR "diseased"[All Fields] OR "illness"[All Fields] OR "illness s"[All Fields] OR "illnesses"[All Fields] OR "wellbeing"[All Fields] OR "health"[MeSH Terms] OR "health"[All Fields] OR "well"[All Fields] OR "well being"[All Fields] | 17,117,082 |
| #7 | #1 AND #4 AND #5 AND #6 | ("fossil fuel industry"[All Fields] OR (("fossil fuels"[Supplementary Concept] OR "fossil fuels"[All Fields] OR "fossil fuel"[All Fields] OR "fossil fuels"[MeSH Terms] OR ("fossil"[All Fields] AND "fuels"[All Fields]) OR ("fossil"[All Fields] AND "fuel"[All Fields])) AND ("companies"[All Fields] OR "company"[All Fields] OR "company s"[All Fields] OR "companys"[All Fields])) OR "coal industry"[All Fields] OR "fossil fuel"[All Fields] OR "oil industry"[All Fields] OR "gas industry"[All Fields] OR "coal mine"[All Fields] OR "coalmine"[All Fields] OR "fracking"[All Fields] OR "gas flaring"[All Fields] OR "oil field"[All Fields] OR "gasworks"[All Fields] OR "coal mining"[All Fields] OR "extractive industry"[All Fields] OR "polluting industry"[All Fields] OR "petrochemical"[All Fields] OR "oil and gas"[All Fields]) AND (("advocacies"[All Fields] OR "advocacy"[All Fields] OR "advocacy s"[All Fields] OR ("policy"[MeSH Terms] OR "policy"[All Fields] OR "policies"[All Fields] OR "policy s"[All Fields]) OR ("strategie"[All Fields] OR "strategies"[All Fields] OR "strategy"[All Fields] OR "strategy s"[All Fields]) OR ("intervention s"[All Fields] OR "interventions"[All Fields] OR "interventive"[All Fields] OR "methods"[MeSH Terms] OR "methods"[All Fields] OR "intervention"[All Fields] OR "interventional"[All Fields]) OR ("campaign"[All Fields] OR "campaign s"[All Fields] OR "campaigned"[All Fields] OR "campaigner"[All Fields] OR "campaigners"[All Fields] OR "campaigning"[All Fields] OR "campaigns"[All Fields]) OR "litigat*"[All Fields] OR "legal action"[All Fields] OR "activism"[All Fields] OR ("action"[All Fields] OR "action s"[All Fields] OR "actions"[All Fields])) AND ("structural"[All Fields] OR "structurally"[All Fields] OR "structurals"[All Fields] OR "structuration"[All Fields] OR "structurations"[All Fields] OR "structure"[All Fields] OR "structure s"[All Fields] OR "structured"[All Fields] OR "structures"[All Fields] OR "structuring"[All Fields] OR ("political"[All Fields] OR "politically"[All Fields] OR "politics"[MeSH Terms] OR "politics"[All Fields] OR "politic"[All Fields]) OR "cultural safety"[All Fields] OR "anti racis*"[All Fields] OR "decolonial*"[All Fields] OR "equit*"[All Fields] OR ("justice s"[All Fields] OR "social justice"[MeSH Terms] OR ("social"[All Fields] AND "justice"[All Fields]) OR "social justice"[All Fields] OR "justice"[All Fields] OR "justices"[All Fields]) OR "rights-based"[All Fields] OR "coproduc*"[All Fields] OR "co produc*"[All Fields] OR ("collective"[All Fields] OR "collectiveness"[All Fields] OR "collectives"[All Fields] OR "collectivities"[All Fields] OR "collectivity"[All Fields]) OR "action research"[All Fields] OR "participatory research"[All Fields] OR "participatory action research"[All Fields] OR "youth participatory action research"[All Fields])) AND ((((((((((((((((("Youth-led"[All Fields] OR "Youth-led"[All Fields] OR "Young people"[All Fields] OR "young person"[All Fields] OR ("adolescent"[MeSH Terms] OR "adolescent"[All Fields] OR "youth"[All Fields] OR "youths"[All Fields] OR "youth s"[All Fields]) OR "adolescen*"[All Fields] OR ("child"[MeSH Terms] OR "child"[All Fields] OR "children"[All Fields] OR "child s"[All Fields] OR "children s"[All Fields] OR "childrens"[All Fields] OR "childs"[All Fields])) AND "Youth involvement"[All Fields]) OR "youth engage*"[All Fields] OR "youth participat*"[All Fields] OR "youth empowerment"[All Fields] OR "youth mobilization"[All Fields] OR (("adolescent"[MeSH Terms] OR "adolescent"[All Fields] OR "youth"[All Fields] OR "youths"[All Fields] OR "youth s"[All Fields]) AND ("mobilisation"[All Fields] OR "mobilisations"[All Fields] OR "mobilise"[All Fields] OR "mobilised"[All Fields] OR "mobiliser"[All Fields] OR "mobilisers"[All Fields] OR "mobilises"[All Fields] OR "mobilising"[All Fields] OR "mobilization"[All Fields] OR "mobilizations"[All Fields] OR "mobilize"[All Fields] OR "mobilized"[All Fields] OR "mobilizer"[All Fields] OR "mobilizers"[All Fields] OR "mobilizes"[All Fields] OR "mobilizing"[All Fields])) OR "youth experience"[All Fields] OR "children and young people"[All Fields] OR "child-led"[All Fields] OR "child-led"[All Fields] OR "child involvement"[All Fields] OR "child-centred"[All Fields] OR "child-centred"[All Fields] OR "youth-informed"[All Fields] OR "child informed"[All Fields] OR "primary school*"[All Fields] OR "secondary school*"[All Fields] OR ("high"[All Fields] AND "school*"[All Fields] AND ("health"[MeSH Terms] OR "health"[All Fields] OR "health s"[All Fields] OR "healthful"[All Fields] OR "healthfulness"[All Fields] OR "healths"[All Fields]))) AND (("health"[MeSH Terms] OR "health"[All Fields] OR "health s"[All Fields] OR "healthful"[All Fields] OR "healthfulness"[All Fields] OR "healths"[All Fields]) AND ("outcome"[All Fields] OR "outcomes"[All Fields]))) AND "or"[All Fields]) AND ("child health"[MeSH Terms] OR ("child"[All Fields] AND "health"[All Fields]) OR "child health"[All Fields])) AND "or"[All Fields]) AND ("public health"[MeSH Terms] OR ("public"[All Fields] AND "health"[All Fields]) OR "public health"[All Fields])) AND "or"[All Fields]) AND (("health"[MeSH Terms] OR "health"[All Fields] OR "health s"[All Fields] OR "healthful"[All Fields] OR "healthfulness"[All Fields] OR "healths"[All Fields]) AND "inequalit*"[All Fields])) AND "or"[All Fields]) AND ("mental health"[MeSH Terms] OR ("mental"[All Fields] AND "health"[All Fields]) OR "mental health"[All Fields])) AND "or"[All Fields]) AND (("health"[MeSH Terms] OR "health"[All Fields] OR "health s"[All Fields] OR "healthful"[All Fields] OR "healthfulness"[All Fields] OR "healths"[All Fields]) AND "inequit*"[All Fields])) AND "or"[All Fields]) AND (("health"[MeSH Terms] OR "health"[All Fields] OR "health s"[All Fields] OR "healthful"[All Fields] OR "healthfulness"[All Fields] OR "healths"[All Fields]) AND "disparit*"[All Fields])) OR ("disease"[MeSH Terms] OR "disease"[All Fields] OR "diseases"[All Fields] OR "disease s"[All Fields] OR "diseased"[All Fields]) OR ("illness"[All Fields] OR "illness s"[All Fields] OR "illnesses"[All Fields]) OR "wellbeing"[All Fields] OR ("health"[MeSH Terms] OR "health"[All Fields] OR "well"[All Fields] OR "well being"[All Fields])) | 997 |
