# Supplementary material for: Interventions involving young people for health equity targeting the fossil fuel industry’s practices and products: A realist review protocol
Source: PLoS One. 2026 Aug 13;21(8):e0356102. doi: 10.1371/journal.pone.0356102 (PMC13472360; doi:10.1371/journal.pone.0356102)
Supplement: Supplementary file 3 [file pone.0356102.s003.docx]

# Candidate Initial Programme Theories (cIPTs) Shared with Expert Steering Group for Feedback

*Instructions: Please provide any comments/suggestions you have on the following theories. This may include (but is not limited to) which aspects are important or missing or need further clarification. Please feel free to email me at* [abi.deivanayagam@ucl.ac.uk](mailto:abi.deivanayagam@ucl.ac.uk) *if you have any further questions or if you would prefer to schedule a short chat to provide any feedback verbally.*

**Programme/intervention** refers to advocacy, litigation, activism, campaigns, policymaking, or strategies that involve young people to address the fossil fuel industry’s role in driving health inequities. Interventions are related to health, with reference to racism. Examples may include youth/health tribunals, local or national campaigns undertaken with youth involvement, or anti-racist clean air campaigns with school children.

**cIPT1:** If health workers challenging the fossil fuel industry use multiple and accessible avenues to communicate with minoritised young people (print, social media, group meetings, etc.) about the intervention on a regular basis with messaging that is relatable, then the messaging will be easy to understand. This will create multiple opportunities for young people members to learn about and engage with the intervention. These approachable and accessible avenues of communication in addition to the young people’s awareness of the intervention enables them to participate in a reciprocal dialogue with the health workers.

**cIPT2:** If the health workers actively listen and respond to minoritised young people’s needs and feedback by modifying the intervention (throughout its implementation) and are transparent about how young people’s feedback has influenced the intervention, then the young people (and their families) will be more motivated to engage and participate in the intervention because it will be relevant to their needs and concerns and they will feel heard by the researchers/implementers because their concerns have been heard and responded to.

**cIPT3:** If the intervention places young people at the centre of the program by involving youth members early and continuously (for example through with local youth on design) then the programme will be appropriate to the local setting’s needs/contexts and will encourage youth and their families to participate in local action because the intervention is being delivered with/by their peers.

**cIPT4:** If health workers acknowledge hierarchies and racism (for example, by using culturally safe and appropriate language, openly discussing power), then this will foster a sense of trust and respect between the young people and the implementers and encourage minoritised youth to engage further with the programme.

**cIPT5:** If young people are providing with both resources (material, skills and human) and agency to make creative public campaigns, then this fosters a sense of empowerment to take action they deem appropriate. The creative campaigns can then influence the general public and shift the status quo, placing more pressure on politicians to act (response).

**cIPT6:** If children’s rights are placed at the centre of the intervention, then these may be used by the implementers as a legal basis to act on health inequities affecting minoritised people driven by the fossil fuel industry.
