# Supplementary material for: Interventions involving young people for health equity targeting the fossil fuel industry’s practices and products: A realist review protocol
Source: PLoS One. 2026 Aug 13;21(8):e0356102. doi: 10.1371/journal.pone.0356102 (PMC13472360; doi:10.1371/journal.pone.0356102)
Supplement: Supplementary file 4 [file pone.0356102.s004.docx]

**Flow chart of realist review search. Version 1.**

Records identified through citations and hand searches of studies and grey literature:

***(N* = )**

Records identified through database and grey literature searches: ***(N* = )**

Database 1 (n= )

Database 2 (n= )

etc.

Grey literature (n =)

Records remaining after deduplication screened against inclusion criteria: title and abstract screening

(n = )

Potential records relevant to the review screened for relevance, richness and rigour: full text screening

(n = )

Records excluded based on title and abstract not meeting inclusion criteria:

(n = )

Records included for synthesis

(n= )

**Any study meeting the initial inclusion criteria**

**Identification**

**Screening & appraisal**

**Included**

Records excluded based on full-text having:

- little to no relevance to development of the initial rough programme theory (n = )

- little to no content that can add to theory development (not conceptually rich)

(n= )

-data are less trustworthy and credible (not sufficiently rigorous)

(n = )

Duplicate records excluded (n = )
