# Supplementary material for: Interventions involving young people for health equity targeting the fossil fuel industry’s practices and products: A realist review protocol
Source: PLoS One. 2026 Aug 13;21(8):e0356102. doi: 10.1371/journal.pone.0356102 (PMC13472360; doi:10.1371/journal.pone.0356102)
Supplement: Supplementary file 5 [file pone.0356102.s005.docx]

**Quality appraisal form**

**Version 1**

**A. REMOVE ALL DUPLICATES ON ENDNOTE**

**B. IMPORT ALL REFERENCES TO RAYYAN**

**C. SCREEN**

Two reviewers will screen each record.

1. Document ID (chronologically i.e. 1, 2, etc.)
2. Details
   2.1 Author name(s)
   2.2 Year published
3. Does the study meet the inclusion criteria?

See inclusion criteria in main text.

**D. QUALITY APPRAISAL – FULL TEXT SCREENING FOR RELEVANCE**

4. Relevance

5.1 Does the evidence address the research questions we are trying to answer?

| High relevance* | - Relates to action taken by an individual or group of people challenging the fossil fuel industry’s practices or products. - Refers to health inequity, specifically due to gender, racial, disability or class injustice. - Describes how adolescents are involved in design, implementation and/or evaluation. - Describes the resources available or provided to practitioners to involve young people or for young people to lead. - Describes the perspectives of young people and the factors affecting their involvement. - Relates to structural racism in policy and decision making. |
| --- | --- |
| Moderate relevance* | - Relates to young people and their involvement in climate justice-related activities. - Describes the health harms of the fossil fuel industry or the health benefits for acting. - Relates to addressing the health impacts of climate change in young people. - Refers to health inequity at the spatial level or other. |
| Low relevance | - Relates to young people’s involvement in climate change-related actions more broadly. - Describes the health impacts of climate change in general |
| No relevance | Does not meet any of the above criteria. |

*Articles ranking high and moderate will be included in the next stage.

**E. QUALITY APPRAISAL - FULL TEXT SCREENING FOR RICHNESS AND RIGOUR**

5. Richness

6.1 Does the evidence provide rich descriptions of concepts and theories about how the intervention works to guide theory development?

| Conceptually rich* | Papers with well-grounded and clearly described theories and concepts |
| --- | --- |
| Conceptually thick* | Papers with a rich description of an intervention was provided, but without explicit reference to the theory underpinning it |
| Conceptually thin | Papers with weak intervention descriptions where discerning theory would have been problematic |

*Articles ranking rich and thick will be included in the next stage.

6. Rigour

7.1 Does the research support the conclusions drawn from it by the researchers?

7.2 Are the data trustworthy and credible?

7. Outcome

| Outcome | Criteria |
| --- | --- |
| Include | Papers in this category have a CMO component to them. Excerpts from the papers have sufficient content that can add to theory development. Data are trustworthy and credible. |
| Maybe | Papers in this category have a CM or CO or MO component to them. Excerpts from the papers have moderate content that can add to theory development. Data are trustworthy and credible. |
| Exclude | Papers in this category have a CM or CO or MO component to them. Although relevant to the research questions, excerpts from the papers have little to no content that can add to theory development. Data are less trustworthy and. |
