# Supplementary material for: Interventions involving young people for health equity targeting the fossil fuel industry’s practices and products: A realist review protocol
Source: PLoS One. 2026 Aug 13;21(8):e0356102. doi: 10.1371/journal.pone.0356102 (PMC13472360; doi:10.1371/journal.pone.0356102)
Supplement: Supplementary file 6 [file pone.0356102.s006.docx]

**Data extraction form**

**Version 1**

1. Document ID (chronologically i.e. 1, 2, etc.)
2. Person extracting
3. Authors
   a. Name(s)
   b. Year published
4. Document type (empirical study (qualitative or quantitative) OR grey literature i.e. report, policy statement, brief, conference proceeding OR legal document)
5. Intervention
   a. Aim
   b. Setting
   c. Delivery mode
   d. Staff
   e. Duration
   f. Details
   g. Degree of involvement of adolescents
6. Sample
   a. Size
   b. Sampling method
7. Structural racism lens
   a. Does the study consider structural racism?
   b. How?
   c. Notes on other equity considerations
8. Context (under what circumstances)
   a. Geographic setting
      i. Country
      ii. City/town (if applicable)
   b. Target population
9. Mechanisms (how)
   a. Page number from which mechanism was drawn
   b. First few words of relevant quotes
10. Outcomes (for whom)
    a. Measures
    b. Time frame
    c. Impact on whom?
    d. Other findings
11. Other notes
    a. Context notes
    b. Mechanism notes
    c. Author’s reflections
    d. Conclusions
    e. Reference
    f. Link
