# Supplementary material for: Interventions involving young people for health equity targeting the fossil fuel industry’s practices and products: A realist review protocol
Source: PLoS One. 2026 Aug 13;21(8):e0356102. doi: 10.1371/journal.pone.0356102 (PMC13472360; doi:10.1371/journal.pone.0356102)
Supplement: Supplementary file 8 [file pone.0356102.s009.docx]

Dear Editors and Reviewer,

We thank the journal and the reviewer for their thoughtful and encouraging review. We are pleased that you found the protocol well-structured, and we appreciate your positive reflections on our realist approach, planned methods, engagement with the Youth Advisory Board and Expert Steering Group, and explicit mention of structural racism and health equity.

Below is a response to each comment, with reference to revisions to the main text.

Yours sincerely,

Dr Abi Deivanayagam

On behalf of co-authors

| Reviewer comment | Response |
| --- | --- |
| While the protocol is strong overall, a few areas would benefit from further clarification. The definition of “intervention” is quite broad and includes advocacy, campaigns, activism, litigation, policymaking, and educational activities. Additional clarification regarding what will constitute a discrete intervention for inclusion in the review would improve transparency and consistency during study selection. | We thank the reviewer for this comment. The definition has been kept broad at this stage because preliminary searches indicated a lack of published literature on actions that involve adolescents in countering the fossil fuel industry’s products and practices, which also have reference to health and wellbeing. The Youth Advisory Board and Expert Steering Group also felt that keeping it broad will help obtain as much data as possible. During a workshop, they listed several types of interventions, and those included in the protocol emerged from the preliminary search and discussion with all study partners.  Furthermore, on page 6, line 214-215, we state “Interventions that are participatory, anti-racist, or rooted in social movements will be prioritised in the search. Studies referencing health inequities linked to racism will rank higher in relevance during appraisal.”  We hope this provides sufficient transparency about which interventions will be prioritised during study selection. |
| The manuscript would also benefit from a clearer explanation of how the structural racism lens will be operationalised during data extraction and synthesis. Providing more detail on the analytical framework or criteria that will be used to identify and assess structural racism within the literature would strengthen the methodology. | We thank the reviewer for this comment. As per the data extraction form, there are three questions to help generate ideas on structural racism for theory-building and refinement.   1. Does the study consider structural racism? 2. How? Is it explicit? 3. Notes on other equity considerations   During data extraction, limitations will be noted e.g. if the describes disparities without explicitly naming racism. Furthermore, page 6, line 215 states “Studies referencing health inequities linked to racism will rank higher in relevance during appraisal.”  Structural racism as a concept will form a core part of synthesis because candidate initial programme theories 1 and 4 explicitly refer to racism or anti-racism. This means that the concept is already embedded in what will become the refined theory. However, we accept that referring to an analytical framework will strengthen the methodology. Therefore, we have added reference to Devakumar and colleagues’ conceptual framework on racism and health (2022). This model is highly suited to this research question because it attributes health inequities to macro-level power hierarchies embedded across society, including institutions, commercial entities, and communities. Applying this model allows us to frame the health harms of fossil fuels as an intergenerational health crisis, driven by racism and extractivism.  See amendment on page 6, lines 217-221. |
| Further clarification regarding the application of Hart’s Ladder of Participation would also be useful, particularly in situations where studies provide limited information about the level of youth involvement. | While we appreciate this comment about Hart's ladder, the search and appraisal criteria will only allow the inclusion of studies that are (1) relevant to the research question(s) and (2) contain rich descriptions of concepts and ideas, that will contribute to theory building (See Table 3 for criteria ranking relevance and S5 File for the quality appraisal form). Therefore, it will not be possible for an article with limited information about youth involvement to be in the final list of papers to extract data from. |
| Similarly, the exclusion criterion relating to climate change interventions that are not specific to the fossil fuel industry may require additional explanation, as many climate-related initiatives may indirectly challenge fossil fuel dependence. | To make this clearer, we have amended the exclusion criterion as follows in Table 2:  “Interventions that are broadly related to climate change, and not specific to countering the fossil fuel industry’s products and practices directly or indirectly.”  We have also added the following on in the “Defining the review scope” section on page 5, line 189:  “[…] and build collective power.”  Please also note that this is addressed on page 5, line 184 on c*orporate political activity (CPA).* In this review, we will include interventions that counter industry CPA both directly and indirectly. This means including, for example, campaigns advocating for clean air, renewable energy initiatives, or education programmes that raise awareness of the industry’s harms. Countering the narratives and sociopolitical norms “framed” by the industry generally requires the intervention to generate collective rather than individual change. |
| There are a few minor editorial issues. In the abstract, the term “youth-led initiates” appears to be a typographical error and should be corrected to “youth-led initiatives.” | Thank you for pointing this out. This correction has been made. |
| The manuscript also alternates between the terms adolescents, youth, and young people; a brief clarification of terminology may improve consistency. | We thank the reviewer for highlighting this. Youth (15-24 years), adolescents (10-19 years) and young people (definition varies across settings) all have different definitions. We have amended the term “young people” to “adolescents” throughout the manuscript to clarify the population we focus on in this review.  The manuscript title remains “young people” as this is widely regarded an umbrella term that includes adolescents (10-19 years). We believe that a title including “young people” instead of “adolescents” may capture more readers at the outset. The term “adolescents” is defined in the introduction section. |
