# Supplementary material for: Interventions involving young people for health equity targeting the fossil fuel industry’s practices and products: A realist review protocol
Source: PLoS One. 2026 Aug 13;21(8):e0356102. doi: 10.1371/journal.pone.0356102 (PMC13472360; doi:10.1371/journal.pone.0356102)
Supplement: Peer review history

# Peer review: Decision Letter 0

**DOI:** 10.1371/journal.pone.0356102.r001

**Authors:** Katherine Kokkinias

8 Jun 2026

Dear Dr. Deivanayagam,

Thank you for submitting your manuscript to PLOS ONE. After careful consideration, we feel that it has merit but does not fully meet PLOS ONE’s publication criteria as it currently stands. Therefore, we invite you to submit a revised version of the manuscript that addresses the points raised during the review process.

==============================

The study protocol has been evaluated by one reviewer, and their comments are available below.

The reviewer raised a number of concerns that need attention. They requested additional clarification on inclusion criteria for a discrete intervention, how structural racism will be operationalized, how Hart’s Ladder of Participation will be applied, and exclusion criteria related to non-fossil fuel related climate change interventions. They have also identified some typographical errors that should be corrected.

Could you please revise the manuscript to carefully address the concerns raised?

==============================

Please submit your revised manuscript by Jul 20 2026 11:59PM. If you will need more time than this to complete your revisions, please reply to this message or contact the journal office at plosone@plos.org. When you're ready to submit your revision, log on to https://www.editorialmanager.com/pone/ and select the 'Submissions Needing Revision' folder to locate your manuscript file.

- A letter that responds to each point raised by the academic editor and reviewer(s). You should upload this letter as a separate file labeled 'Response to Reviewers'.
- A marked-up copy of your manuscript that highlights changes made to the original version. You should upload this as a separate file labeled 'Revised Manuscript with Track Changes'.
- An unmarked version of your revised paper without tracked changes. You should upload this as a separate file labeled 'Manuscript'.

If you would like to make changes to your financial disclosure, please include your updated statement in your cover letter. Guidelines for resubmitting your figure files are available below the reviewer comments at the end of this letter.

If applicable, we recommend that you deposit your laboratory protocols in protocols.io to enhance the reproducibility of your results. Protocols.io assigns your protocol its own identifier (DOI) so that it can be cited independently in the future. For instructions see: https://journals.plos.org/plosone/s/submission-guidelines#loc-laboratory-protocols. Additionally, PLOS ONE offers an option for publishing peer-reviewed Lab Protocol articles, which describe protocols hosted on protocols.io. Read more information on sharing protocols at https://plos.org/protocols?utm_medium=editorial-email&utm_source=authorletters&utm_campaign=protocols.

As the corresponding author, your ORCID iD is verified in the submission system and will appear in the published article. PLOS supports the use of ORCID, and we encourage all coauthors to register for an ORCID iD and use it as well. Please encourage your coauthors to verify their ORCID iD within the submission system before final acceptance, as unverified ORCID iDs will not appear in the published article. *Only*  the individual author can complete the verification step; PLOS staff *cannot*  verify ORCID iDs on behalf of authors.

We look forward to receiving your revised manuscript.

Kind regards,

Katherine Demi Kokkinias, Ph.D.

Staff Editor

PLOS One

Journal Requirements:

When submitting your revision, we need you to address these additional requirements.

1. Please ensure that your manuscript meets PLOS ONE's style requirements, including those for file naming. The PLOS ONE style templates can be found at

https://journals.plos.org/plosone/s/file?id=wjVg/PLOSOne_formatting_sample_main_body.pdf and

https://journals.plos.org/plosone/s/file?id=ba62/PLOSOne_formatting_sample_title_authors_affiliations.pdf

2. Thank you for stating the following financial disclosure:

“TAD declares being funded by the National Institute for Health Research (NIHR) Doctoral Fellowship (NIHR303268) focusing on fossil fuels, climate change, racism, and health inequalities.

MvS’s position is supported by the National Institute for Health and Care Research (grant number NIHR156820 Addressing the Commercial Determinants of Health in Sub-Saharan Africa) and by the Centre for Pesticide Suicide Prevention which is funded by the Good Ventures Foundation at the recommendation of Open Philanthropy.

MvS has funding through the SPECTRUM consortium which is funded by the UK Prevention Research Partnership (UKPRP), a consortium of UK funders [UKRI Research Councils: Medical Research Council (MRC), Engineering and Physical Sciences Research Council (EPSRC), Economic and Social Research Council (ESRC) and Natural Environment Research Council (NERC); Charities: British Heart Foundation, Cancer Research UK, Wellcome and The Health Foundation; Government: Scottish Government Chief Scientist Office, Health and Care Research Wales, National Institute of Health Research (NIHR) and Public Health Agency (NI)].”

Please state what role the funders took in the study.  If the funders had no role, please state: "The funders had no role in study design, data collection and analysis, decision to publish, or preparation of the manuscript."

If this statement is not correct you must amend it as needed.

Please include this amended Role of Funder statement in your cover letter; we will change the online submission form on your behalf.

3. Please provide a complete Data Availability Statement in the submission form, ensuring you include all necessary access information or a reason for why you are unable to make your data freely accessible. If your research concerns only data provided within your submission, please write "All data are in the manuscript and/or supporting information files" as your Data Availability Statement.

4. If the reviewer comments include a recommendation to cite specific previously published works, please review and evaluate these publications to determine whether they are relevant and should be cited. There is no requirement to cite these works unless the editor has indicated otherwise.

[Note: HTML markup is below. Please do not edit.]

Reviewers' comments:

Reviewer's Responses to Questions

**Comments to the Author**

1. Does the manuscript provide a valid rationale for the proposed study, with clearly identified and justified research questions?

Reviewer #1: Yes

**********

2. Is the protocol technically sound and planned in a manner that will lead to a meaningful outcome and allow testing the stated hypotheses?

Reviewer #1: Yes

**********

3. Is the methodology feasible and described in sufficient detail to allow the work to be replicable?

Reviewer #1: Yes

**********

4. Have the authors described where all data underlying the findings will be made available when the study is complete?

The PLOS Data policy

Reviewer #1: Yes

**********

5. Is the manuscript presented in an intelligible fashion and written in standard English?

Reviewer #1: Yes

**********

Please use the space provided to explain your answers to the questions above and, if applicable, provide comments about issues authors must address before this protocol can be accepted for publication. You may also include additional comments for the author, including concerns about research or publication ethics.

You may also provide optional suggestions and comments to authors that they might find helpful in planning their study.

Reviewer #1: The manuscript presents a timely and important realist review protocol addressing interventions involving young people in countering the health harms and inequities associated with the fossil fuel industry. The topic is highly relevant and addresses an important gap in the literature at the intersection of climate change, public health, health equity, youth participation, and the commercial determinants of health.

Overall, the protocol is well written, logically structured, and grounded in established realist review methodology. The rationale for the study is clearly presented, and the research questions are appropriate and aligned with the stated objectives. The use of a realist review approach is well justified, as it is particularly suited to understanding how complex interventions work, for whom they work, and under what circumstances they are effective.

A major strength of the protocol is the involvement of a Youth Advisory Board and an Expert Steering Group throughout the review process. This participatory approach enhances the relevance and credibility of the proposed review and is consistent with the manuscript's emphasis on youth engagement and health equity. The explicit consideration of structural racism and health inequities also adds important value to the proposed work.

The methodology is generally sound and follows recognised RAMESES standards. The search strategy is comprehensive and includes both academic and grey literature sources. The planned use of Context-Mechanism-Outcome (CMO) configurations is appropriate for theory development and refinement.

While the protocol is strong overall, a few areas would benefit from further clarification. The definition of “intervention” is quite broad and includes advocacy, campaigns, activism, litigation, policymaking, and educational activities. Additional clarification regarding what will constitute a discrete intervention for inclusion in the review would improve transparency and consistency during study selection.

The manuscript would also benefit from a clearer explanation of how the structural racism lens will be operationalised during data extraction and synthesis. Providing more detail on the analytical framework or criteria that will be used to identify and assess structural racism within the literature would strengthen the methodology.

Further clarification regarding the application of Hart’s Ladder of Participation would also be useful, particularly in situations where studies provide limited information about the level of youth involvement. Similarly, the exclusion criterion relating to climate change interventions that are not specific to the fossil fuel industry may require additional explanation, as many climate-related initiatives may indirectly challenge fossil fuel dependence.

There are a few minor editorial issues. In the abstract, the term “youth-led initiates” appears to be a typographical error and should be corrected to “youth-led initiatives.” The manuscript also alternates between the terms adolescents, youth, and young people; a brief clarification of terminology may improve consistency.

In conclusion, this is a well-conceived and methodologically appropriate protocol addressing an important and underexplored area of public health research. Subject to minor clarification of several methodological aspects, I believe the manuscript is suitable for publication.

**********

what does this mean?). If published, this will include your full peer review and any attached files.

If you choose “no”, your identity will remain anonymous but your review may still be made public.

**Do you want your identity to be public for this peer review?**  For information about this choice, including consent withdrawal, please see our Privacy Policy

Reviewer #1: **Yes:** Dr Sandhya Ahuja, Sr Public Health Expert, Information Management and Analysis

**********

[NOTE: If reviewer comments were submitted as an attachment file, they will be attached to this email and accessible via the submission site. Please log into your account, locate the manuscript record, and check for the action link "View Attachments". If this link does not appear, there are no attachment files.]

To ensure your figures meet our technical requirements, please review our figure guidelines: https://journals.plos.org/plosone/s/figures

You may also use PLOS’s free figure tool, NAAS, to help you prepare publication quality figures: https://journals.plos.org/plosone/s/figures#loc-tools-for-figure-preparation.

NAAS will assess whether your figures meet our technical requirements by comparing each figure against our figure specifications.

# Author comment: Author response to Decision Letter 1

**DOI:** 10.1371/journal.pone.0356102.r002

25 Jun 2026

Please see the attached document for responses to reviewer and editor comments.

Attachment Submitted filename: Response to reviewers_250626.docx

# Peer review: Decision Letter 1

**DOI:** 10.1371/journal.pone.0356102.r003

**Authors:** Tingzhen Ming

29 Jul 2026

Interventions involving young people for health equity targeting the fossil fuel industry's practices and products: a realist review protocol

PONE-D-26-08528R1

Dear Dr. Deivanayagam,

We’re pleased to inform you that your manuscript has been judged scientifically suitable for publication and will be formally accepted for publication once it meets all outstanding technical requirements.

Within one week, you’ll receive an e-mail detailing the required amendments. When these have been addressed, you’ll receive a formal acceptance letter and your manuscript will be scheduled for publication.

An invoice will be generated when your article is formally accepted. Please note, if your institution has a publishing partnership with PLOS and your article meets the relevant criteria, all or part of your publication costs will be covered. Please make sure your user information is up-to-date by logging into Editorial Manager at Editorial Manager® and clicking the ‘Update My Information' link at the top of the page. For questions related to billing, please contact billing support.

If your institution or institutions have a press office, please notify them about your upcoming paper to help maximize its impact. If they’ll be preparing press materials, please inform our press team as soon as possible -- no later than 48 hours after receiving the formal acceptance. Your manuscript will remain under strict press embargo until 2 pm Eastern Time on the date of publication. For more information, please contact onepress@plos.org.

Kind regards,

Tingzhen Ming, Ph.D.

Academic Editor

PLOS One

Additional Editor Comments (optional):

Reviewers' comments:

Reviewer's Responses to Questions

**Comments to the Author**

1. Does the manuscript provide a valid rationale for the proposed study, with clearly identified and justified research questions?

Reviewer #1: Yes

**********

2. Is the protocol technically sound and planned in a manner that will lead to a meaningful outcome and allow testing the stated hypotheses?

Reviewer #1: Yes

**********

3. Is the methodology feasible and described in sufficient detail to allow the work to be replicable?

Reviewer #1: Yes

**********

4. Have the authors described where all data underlying the findings will be made available when the study is complete?

The PLOS Data policy

Reviewer #1: Yes

**********

5. Is the manuscript presented in an intelligible fashion and written in standard English?

Reviewer #1: Yes

**********

Please use the space provided to explain your answers to the questions above and, if applicable, provide comments about issues authors must address before this protocol can be accepted for publication. You may also include additional comments for the author, including concerns about research or publication ethics.

You may also provide optional suggestions and comments to authors that they might find helpful in planning their study.

Reviewer #1: Thank you for your careful and thoughtful revision of the manuscript. I appreciate the detailed responses to my previous comments and the revisions made throughout the protocol. The additional clarification regarding the definition of interventions, operationalisation of the structural racism framework, application of Hart's Ladder of Participation, and refinement of the inclusion and exclusion criteria has strengthened the methodological transparency of the protocol. I also appreciate the improvements in terminology and correction of the editorial issues identified during the previous review. I have no further substantive comments and believe the manuscript is suitable for publication.

**********

what does this mean?). If published, this will include your full peer review and any attached files.

If you choose “no”, your identity will remain anonymous but your review may still be made public.

**Do you want your identity to be public for this peer review?**  For information about this choice, including consent withdrawal, please see our Privacy Policy

Reviewer #1: **Yes:** Dr Sandhya Ahuja, Sr Public Health Expert, Information Management and Analysis

**********

# Editor report: Acceptance letter

**DOI:** 10.1371/journal.pone.0356102.r004

**Authors:** Tingzhen Ming

PONE-D-26-08528R1

PLOS One

Dear Dr. Deivanayagam,

I'm pleased to inform you that your manuscript has been deemed suitable for publication in PLOS One. Congratulations! Your manuscript is now being handed over to our production team.

At this stage, our production department will prepare your paper for publication. This includes ensuring the following:

* All references, tables, and figures are properly cited

* All relevant supporting information is included in the manuscript submission,

* There are no issues that prevent the paper from being properly typeset

You will receive further instructions from the production team, including instructions on how to review your proof when it is ready. Please keep in mind that we are working through a large volume of accepted articles, so please give us a few days to review your paper and let you know the next and final steps.

Lastly, if your institution or institutions have a press office, please let them know about your upcoming paper now to help maximize its impact. If they'll be preparing press materials, please inform our press team within the next 48 hours. Your manuscript will remain under strict press embargo until 2 pm Eastern Time on the date of publication. For more information, please contact onepress@plos.org.

You will receive an invoice from PLOS for your publication fee after your manuscript has reached the completed accept phase. If you receive an email requesting payment before acceptance or for any other service, this may be a phishing scheme. Learn how to identify phishing emails and protect your accounts at https://explore.plos.org/phishing.

If we can help with anything else, please email us at customercare@plos.org.

Thank you for submitting your work to PLOS ONE and supporting open access.

Kind regards,

PLOS ONE Editorial Office Staff

on behalf of

Dr. Tingzhen Ming

Academic Editor

PLOS One
